# Burned-out testis tumour that metastasized to retroperitoneal lymph nodes: a case report

**DOI:** 10.1186/1752-1947-3-7266

**Published:** 2009-05-29

**Authors:** Mehmet Yucel, Sahin Kabay, Ugur Saracoglu, Soner Yalcinkaya, Namik Kemal Hatipoglu, Erol Aras

**Affiliations:** 1 Department of Urology, Faculty of Medicine, Dumlupinar University, 43270 Kutahya, Turkey; 2 Department of Urology, Okmeydani Educational Hospital, Istanbul, Turkey

## Abstract

**Introduction:**

Burned-out testicular tumour is a very rare clinical entity. There is no clinical finding in the testicle, because it regresses spontaneously with no treatment, and generally presents with metastases. Abdominal masses in young male patients may sometimes be caused by a metastatic burned-out testicular tumour. We report a patient with a burned-out testicular tumour that metastasized to retroperitoneal lymph nodes.

**Case presentation:**

A 28-year-old man complained of an abdominal mass and continuously increasing pain over the previous 2 months. A midabdominal mass, atrophy and minimal induration in the right testis were revealed on physical examination. Ultrasound findings revealed focally increased echogenicity, which is typical of burned-out tumours. Inguinal orchiectomy was performed, and the histological examination of the biopsy specimen revealed a large area of hyalinization, tubular hyalinization, interstitial fibrosis and focal Leydig cell hyperplasia, with no abnormal pathological findings in the epididymis and spermatic cord. The final pathological diagnosis was concluded as "burned-out" testicular tumour. Surgical treatment was followed by appropriate chemotherapy and in the follow-up, the abdominal mass was observed to regress. The patient is currently free of disease 5 years after diagnosis.

**Conclusion:**

For the detection of intratesticular lesions, especially in patients with extragonadal metastatic involvement and normal palpation findings for the testis, scrotal sonography is very important. A burned-out testicular tumour should be considered and testis biopsies should be performed if there is any risk factor of malignancy.

## Introduction

Burned-out tumour of the testis is a very rare clinical entity. The term 'burned-out' tumour of the testis describes a spontaneously and completely regressed testicular tumour with no treatment. It presents by metastases to the retroperitoneum, mediastinum, lymph nodes, lungs and liver [1].

We report a patient with a burned-out testicular tumour that metastasized to the retroperitoneal area. Testicular germ cell tumours metastasize predominantly to the retroperitoneal lymph nodes and subsequently to the lungs.

## Case presentation

A 28-year-old man complained of an abdominal mass and continuously increasing pain over the previous 2 months. Physical examination revealed a midabdominal mass on abdominal palpation, atrophy and minimal induration in the right testis on scrotal palpation. The left testis was found to be normal. No abnormal finding was noted on digital rectal examination. Examination of the other systems, including the endocrine system, was normal.

**Figure 1:**
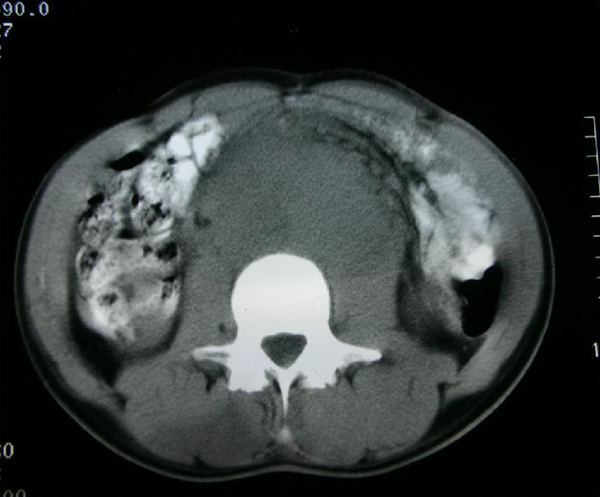
**Abdominal computed tomography images revealing a large retroperitoneal mass, extending across the midline**.

His white blood cell count and urine microscopy were normal. Aspartate aminotransferase (AST): 56 mg/dl (normally 1 to 40), alanine aminotransferase (ALT): 42 mg/dl (normally 1 to 38), alkaline phosphatase (ALP): 768 mg/dl (normally 80 to 306), gamma glutamyl transferase (GGT): 160 mg/dl (normally 6 to 50) and lactate dehydrogenase (LDH): 6302 mg/dl (normally 266 to 501) were found in the blood biochemistry. His erythrocyte sedimentation rate was high (110 mm/hour). Values for alpha-fetoprotein (AFP) (5.9 ng/ml (normally 0 to 10.0)) and beta human chorionic gonadotrophin hormone (β-hCG) (0.773 mIU/ml (normally <4)) were within normal limits. Chest X-ray was normal. Scrotal ultrasonogram revealed minimal hypoechogeneity and non-homogeneity in the right testis. Abdominal computed tomography (CT) revealed a very large retroperitoneal mass (13×8 cm), extending across the midline (Figure 1). Thorax CT was normal.

Needle aspiration biopsy was performed for the retroperitoneal mass and malignant tumour infiltration was reported. After the immunohistochemical studies, cytokeratin (CK) (-), vimentin (-), leukocyte common antigen (LCA) (-), β-hCG (-) and placental alkaline phosphatase (PLAP) (+) immunoreactivity were noted, indicating a germ cell tumour.

**Figure 2:**
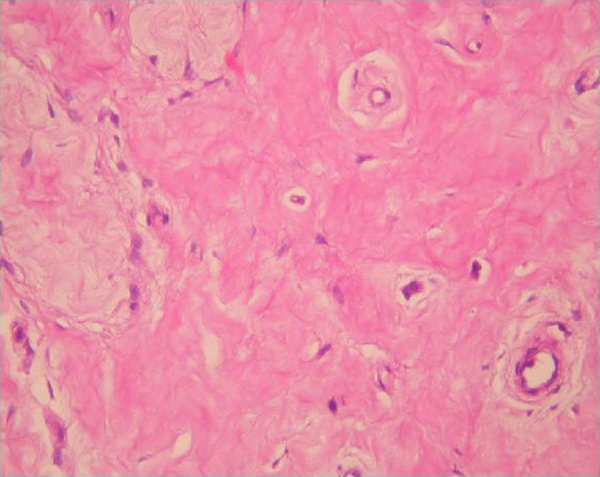
**Histological specimen of the testis showing large hyalinization areas, tubular hyalinization, interstitial fibrosis and focal Leydig cell hyperplasia (burned-out germ cell tumour)**.

Scrotal ultrasonogram revealed hypoechogeneity and non-homogeneity in the right testicle. Evidence of a tumour was not found. The left testicle and epididymis were normal. After pre-operative evaluation, right inguinal orchiectomy was performed to determine the primary site of the tumour. Histological examination of the biopsy specimen revealed a large area of hyalinization, tubular hyalinization, interstitial fibrosis and focal Leydig cell hyperplasia. There were no pathological findings in the epididymis and spermatic cord. The final pathological diagnosis was 'burned-out' testicular tumour (Figure 2).

**Figure 3:**
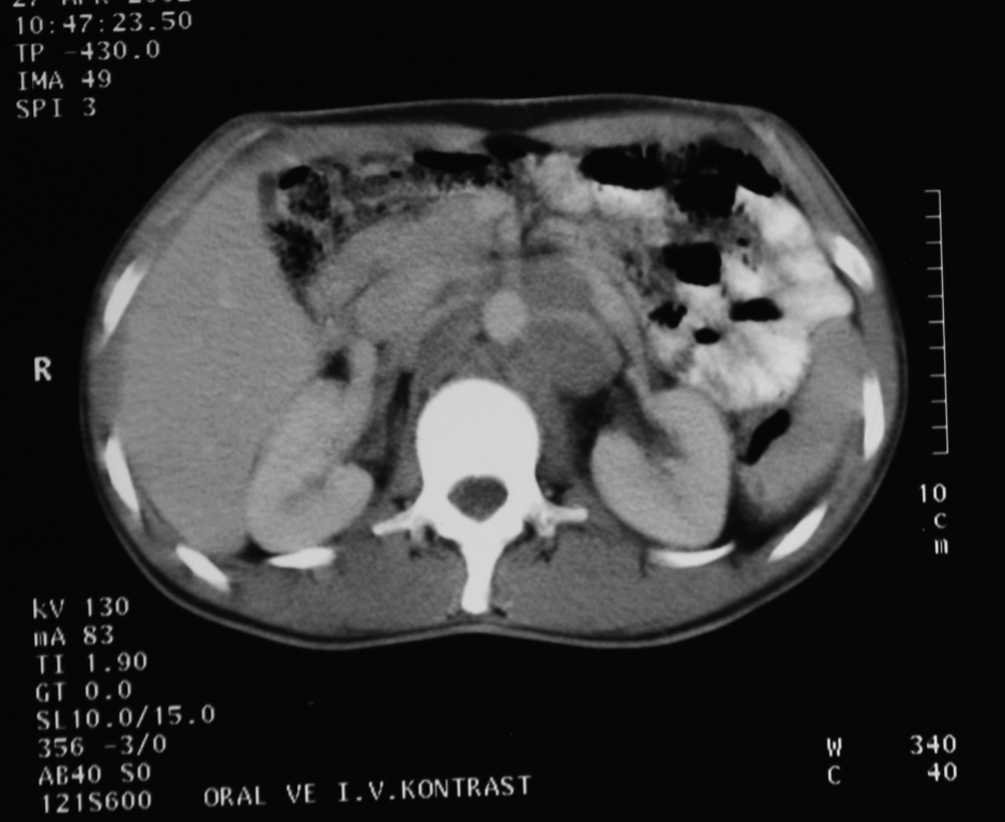
**After the four courses of chemotherapy treatment, computed tomography showed that the abdominal mass had regressed**.

Because of the large retroperitoneal lymph node metastasis, primary chemotherapeutic treatment was performed. Combination chemotherapy, consisting of bleomycin, etoposide and cisplatin, was given in three weekly cycles of four courses. After the four courses of chemotherapy treatment, the abdominal mass had regressed from 13×8 cm to 3×2 cm (Figure 3).

After the chemotherapy, a control CT scan was obtained revealing regression in the para-aortic and para-caval lymph nodes (3×2 cm and multiple lymph nodes). After 6 months, tumour markers had increased (AFP: 3.59 ng/ml (normally 0 to 10.0) and β-hCG: 20.95 mIU/ml (normally <4)). Because only the β-hCG level had increased, the histology of the primary tumour seemed to indicate a seminoma. For this reason, retroperitoneal lymph node dissection was planned. Exploratory laparotomy revealed a retroperitoneal mass which extended both sides of the midline and involved the major vessels and extended to the eosophogastric junction, indicating an unresectable mass. A biopsy was performed from the unresectable mass and histological examination of the biopsy specimen showed only necrotic tissue and no tumour cells.

Salvage chemotherapy consisting of paclitaxel, ifosfamide and cisplatin (TIP) was given in monthly cycles of four courses. After this chemotherapy, an abdominal CT scan revealed regression in the para-aortic and para-caval lymph nodes (milimetrical). AFP and β-hCG levels were normal (AFP: 1 ng/ml (normally 0 to 10.0) and β-HCG: 0.180 mIU/ml (normally <4)).

The patient has been disease and recurrence free for 5 years since the primary surgical and medical treatment.

## Discussion

Extragonadal germ cell tumours are rare but well-known neoplasms. Primary extragonadal germ cell tumours constitute 3% to 5% of all germ cell tumours. More than 60% of these tumours are seminomas arising in the anterior mediastinum and retroperitoneum [2]. Visceral metastases are rarely observed in the lungs, liver, brain, gastrointestinal tract, bones, kidneys, adrenal glands, tonsil, spleen and peritoneum. Metastases to the thyroid and prostate glands have also been reported [3].

In 1927, Prym [4] reported testicular scarring in a patient with an extragonadal tumour at autopsy. Azzopardi *et al.*[5] have showed cell-poor, collagen-rich fibrous scars in some palpably normal testes of patients with metastatic non-seminomatous germ cell tumours. They detected hematoxyphilic deposits in seminiferous tubuli which they called hematoxyphilic bodies. Comiter *et al.*[6] reported diffuse atrophy of the testis with hematoxyphilic bodies and psammoma bodies in some patients. They believed that these bodies represented echogenic foci on sonography.

A burned-out testicular tumour is a spontaneously and completely regressed testicular tumour. It presents by its metastases to the retroperitoneum, mediastinum, lymph nodes, lungs, liver or prostate [2]. Ultrasonographic findings in burned-out tumours are raised echogenicity in a focal area, probably due to calcium deposits and fibrosis. There are no tumoural findings on pathological examination of the testis [7]. Despite ultrasonographic findings being normal, testis biopsies should be performed if there is any suspicion, and/or risk factors for in situ malignancies.

The majority of mediastinal and central nervous system extragonadal germ cell tumours are believed to arise from primary lesions. Retroperitoneal germ cell tumours appear to arise from primary testicular lesions [8]. Primary extragonadal germ cell tumours of the retroperitoneum are probably a rare entity. They should be considered to be metastases of a viable or burned-out testicular cancer until proven otherwise histologically [9].

According to one hypothesis, these tumours arise from primitive totipotential cells during the morula or blastula stage that later transform into germinal cells [10]. This hypothesis considers these extragonadal germ cell tumours to be metastases from a primary testicular tumour. Usually only a fibrous scar can be found on histological examination which presumably represents the regression of the original tumour [8,9].

Fabre *et al.*[11] declared that the clinical presentations of patients with "burned-out" testicular tumours are very variable, and that this diagnosis, although infrequent, must be considered; and extragonadal germ cell tumours should be considered to be metastases of a "burned out" testicular tumour, and must be investigated. They recommended removing the testis although only intratubular germ cell neoplasia is usually found, which does not provide any diagnostic argument.

In the presence of retroperitoneal lymph nodes, a testicular ultrasound examination can detect tiny intratesticular lesions, minimizing the possibility of a primary extragonadal germ cell tumour. It is important to distinguish 'burned out' tumours of the testis from true extragonadal germ cell tumours because primary removal of the testicular tumour is necessary for treatment [12].

Orchiectomy is generally completed with cisplatin-based combination chemotherapy protocols. This therapy is very effective in the treatment of seminomas and non-seminomatous germ cell tumours. We also treated our patient with combination chemotherapy with bleomycin, etoposide and cisplatin, and in a 4-year follow-up, no disease recurrence was observed.

## Conclusion

Scrotal sonography is very important for the detection of intratesticular lesions, especially in patients with extragonadal metastatic involvement and normal palpation findings for the testis. A burned-out testicular tumour should be considered when punctuate echogenic foci are seen without any evidence of hypoechoic mass lesions. Testis biopsies should be performed if there is any risk factor for malignancy.

## Abbreviations

AFP: alpha-fetoprotein; β-hCG: beta human chorionic gonadotrophin hormone; AST: aspartate aminotransferase; ALT: alanine aminotransferase; ALP: alkaline phosphatase; CK: cytokeratin; CT: computed tomography; GGT: gamma glutamyl transpeptidase; LCA: leukocyte common antigen; LDH: lactate dehydrogenase; PLAP: placental alkaline phosphatase.

## Consent

Written informed consent was obtained from the patient for publication of this case report and any accompanying images. A copy of the written consent is available for review by the Editor-in-Chief of this journal.

## Competing interests

The authors declare that they have no competing interests.

## Authors' contributions

MY, US and EA have made substantial contributions to conception and design, acquisition of data, and analysis and interpretation of data. NKH, SY and SK have been involved in drafting the manuscript or revising it critically for important intellectual content. All authors read and approved the final manuscript.
